# An efficient approach based on 3D reconstruction of CT scan to improve the management and monitoring of COVID-19 patients

**DOI:** 10.1016/j.heliyon.2020.e05453

**Published:** 2020-11-07

**Authors:** Mouad Hasni, Zineb Farahat, Azar Abdeljelil, Kamal Marzouki, Mohamed Aoudad, Zakaria Tlemsani, Kawtar Megdiche, Nabil Ngote

**Affiliations:** a Medical Simulation Center of the Cheikh Zaid Foundation, Rabat, Morocco; b Covid-19 Unit, Cheikh Zaid Foundation, Rabat, Morocco; c Abulcasis International University of Health Sciences, Rabat, Morocco

**Keywords:** Biomedical engineering, Infectious disease, Pathology, Radiology, Respiratory system, Virology, SARS-CoV-2, COVID-19, RT-PCR, CT, DICOM, ANATOMAGE table, Diagnostic, Management, Monitoring, Follow-up

## Abstract

**Purpose:**

To reconstruct a 3D visualization from CT images of COVID-19 patients in order to improve understanding of the disease for better management and follow-up.

**Materials and methods:**

We have retrieved CT images of 185 COVID-19 patients from the Cheikh Zaid International University Hospital in Rabat, Morocco. We then performed computer processing that allowed us to obtain a 3D visualization of these patients.

**Results:**

In this article, we have chosen to do 3D reconstruction of three specific cases among 185 patients:

- Cases (A1, A2) which are negative RT-PCR patient with normal CT images.

- Cases (B1, B2) which are positive RT-PCR patient with abnormal CT images.

- Case (C) which is a negative RT-PCR patient with CT abnormalities.

To improve our results and have a better quality of the 3D reconstruction, we used different algorithms and a specific row data processing.

**Conclusion:**

3D reconstruction has a significant role in the diagnosis and management of COVID-19 patients. The quality and reliability of 3D reconstructions allow the clinician to make a quick and efficient diagnosis and avoid an eventual false negative (produced by the RT-PCR test). We suggest including chest 3D reconstruction in the patient management and prognosis evaluation.

## 1 Introduction

During the month of December 2019, in Wuhan County, capital of the Hubei Province in China, several cases of pneumonia linked to a newly identified coronavirus appeared [1]. In February 2020, the World Health Organization (WHO) named the disease COVID-19 [2] and the Coronavirus Study Group (CSG) proposed to name this new coronavirus SARS-CoV-2 [3].

The infection related to this virus has surprised the world by its rapid spread and its potential virulence, profoundly impacting everyone's life on a global scale. In early March 2020, the WHO decided to classify COVID-19 a pandemic due to the rapid increase in the number of cases outside China and the growing number of affected countries [4]. Indeed, as of today (22 May 2020), there are nearly 5,220,000 confirmed cases, spread over 185 countries, with nearly 335,000 deaths, i.e. a mortality rate close to 6.4% [5].

In this context, and considering the absence of specific efficient drugs or vaccines against COVID-19, it becomes imperative to detect the disease at an early stage and immediately isolate the infected person from the healthy population.

To date, the flagship clinical diagnostic method for COVID-19 has been the detection of nucleic acid in nasal and throat swab samples [6]. The confirmation of a COVID-19 positive case is based on RT-PCR (Real-Time Polymerase Chain Reaction) on a nasal swab or nasopharyngeal lavage sample with a sensitivity of 75% and 95% respectively for both sampling methods [7].

Nevertheless, RT-PCR diagnosis during a pandemic, with a massive influx of suspect patients, is constrained by the sensitivity limits of sampling techniques, transportation and routing constraints, the performance of swab kits, and the time required for gene amplification.

In practice, Computed Tomography (CT) scan without the injection of the contrast agent remains a reference examination that allows rapid screening, better sensitivity, estimated at 98% [8], radio-clinical disease evolution monitoring, together with an evaluation of pulmonary sequelae after recovery [9, 10].

Indeed, typical findings in thoracic CT include multifocal bilateral frosted glass aspects with uneven consolidation, prominent peripheral subpleural distribution and a predilection for the preferred posterior or lower lobe [11].

Several recent studies have demonstrated the value of CT scanning for the early detection and management of COVID-19 pulmonary manifestations [12, 13, 14, 15, 16, 17].

Recently, a team of researchers has proposed a new digital diagnostic method with 3D reconstruction [18]. Indeed, the digital tools developed around the medical technologies to which they are connected are revolutionizing the diagnostic approach, teaching methods and scientific research.

The pandemic caused by COVID-19 has anguished researchers and particularly clinicians because of the lack of understanding of the manifestations of this disease. This places 3D reconstruction techniques based on 2D DICOM images at the heart of efforts to better identify the damage caused by the coronavirus.

These techniques should also enable medical personnel to visually observe subtle structures, improve the quality, speed and reliability of diagnosis, increase the cure rate, and reduce mortality and complications related to COVID-19.

To date, little is known about the damage caused by the disease and no direct visualization of its evolution has ever been made. The aim of this paper is therefore to present a realistic 3D reconstruction methodology exploiting the performances of the Anatomage Table [19], after implementing 2D scanner images of patients with COVID-19.

The rest of the paper is organized as follows. Section 2 describes the materials and methods. Section 3 and 4 are respectively devoted to results and discussion. Finally, conclusions are reported in Section 5.

## 2 Materials and methods

### 2.1 Patients and data sources

Our study is a prospective analysis approved by the ethics committee of the Cheikh Zaid International University Hospital under the number CEFCZ/AB/2020/PR09, and patient consent was obtained.

185 patients suspected of COVID-19, on the base of clinical symptomatology or uncontrasted CT scan with suspicious images, were admitted to isolation departments at Cheikh Zaid Hospital in Rabat, since March 16, 2020, with a gender ratio (Male/Female) of 1.22 and mean age of 53 (+/- 18). Details of the clinical characteristics of these patients are summarized in Table 1.

**Table 1:** Clinical Characteristics of COVID-19 and non-COVID-19 patients.

	COVID-19 (n = 90)	Non-COVID-19 (n = 95)
Age (year)		
Mean age	53 (+/-18)	
<20	17 (18.8%)	7 (7.3%)
20-39	28 (31.1%)	21 (22.1)
40-59	34 (37.7)	48 (50.5%)
≥60	11 (12.2%)	19 (20%)
Sex		
Male	53 (58.8%)	49 (51.5%)
Female	37 (41.1%)	46 (48.4%)
Presence of Fever		
Fever	75 (83.3%)	69 (72.6%)
No fever	15 (16.6%)	26 (27.3%)
White blood cell Count		
Elevated	82 (91.1%)	63 (66.3%)
Normal	8 (8.8%)	32 (33.6%)
Lymphocyte count		
Normal	12 (13.3%)	72 (75.7%)
Decreased	78 (86.6%)	23 (24.2%)
Comorbidities		
Cardiovascular Disease	18 (20%)	14 (14.7%)
Hypertension	21 (23.3%)	11 (11.5%)
COPD	7 (7.7%)	8 (8.4%)
Dibetes	12 (13.3%)	9 (9.4%)
Chronic liver Disease	0 (0%)	0 (0%)
Chronic kidney Disease	2 (2.2%)	1 (1%)
Malignant tumor	4 (4.4%)	0 (0%)
HIV	0 (0%)	0 (0%)
Severity		
Mild	52 (57.7%)	-
Medium	14 (15.5%)	-
Severe	15 (16.3%)	-
Critical	9 (10%)	-

After RT-PCR test was performed, 38 patients came back positive, and 52 PCR negative patients were declared positive based on their CT images. All patients declared positive were treated for a minimum of 10 days according to the Moroccan ministry of health protocols.

### 2.2 CT image acquisition

All images were obtained on one CT system (Somatom Def AS, Siemens Healthineers, Germany) with patients in supine position. The main scanning parameters were as follow: Tube voltage: 120 kV, pitch factor = 0.3–1.5 mm, recon matrix = 512 x 512, slice thickness = 1 mm.

### 2.3 Review of CT images

All digital imaging and communication in medicine from cases seen at the Cheikh Zaid International University Hospital were analyzed and reviewed by three radiologists, who overlooked to RT-PCR results. They decided on positive or negative CT finding by consensus. The epidemiological history and clinical symptoms (fever and dry cough) were available for all readers. The radiologists classified chest CT as positive or negative for COVID-19.

### 2.4 3D reconstruction of CT images

First, to have a better 3D reconstruction, we directly extracted the DICOM files raw data from the imaging modality console. Then, we anonymized the extracted 2D DICOM images using Matlab software, in order to ensure the hospital's best privacy to the patient.

To open these files and have a better 3D reconstruction's quality on the TableEDU 4.0 software, we deleted the corrupted files. Then, an adapted data processing was done on a personal computer, before transferring the DICOM files to the Anatomage table [19, 20, 21, 22, 23, 24].

**Figure 1:**
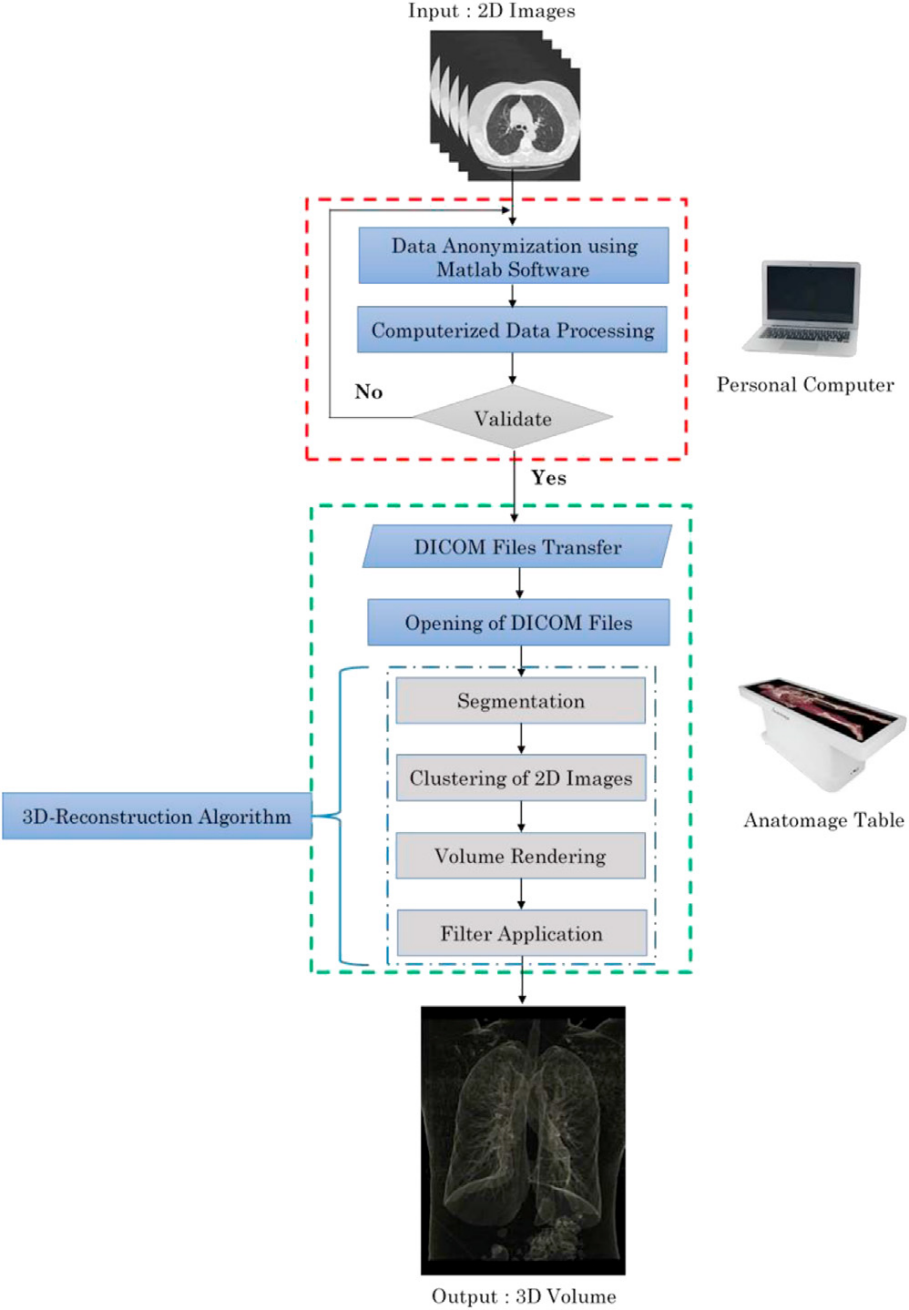
Flowchart of the 3D reconstruction realization.

At last, a 3D reconstruction algorithm was used on the TableEDU 4.0 software, as shown in the flowchart bellow (Figure 1). It enabled users to display a dissectible 3D volume on the Anatomage table's screen. The 3D reconstructions are obtained using image segmentation and contour detection provided automatically by the TableEDU 4.0 software of the Anatomage table. This allows to stack images with the same gray scale. The obtained clusters represent a 3D volume that can be adjusted using different filters of the software. The 3D volume can be modified using different tools such as the volume visibility control tool and the contrast slider tool.

## 3 Results

This study included 185 patients. The main age is 53 (+/- 18) and there were 102 males (55.1%) and 83 females (44.8%).

Only 38 patients (20.5%) were declared positive based on the results of the RT-PCR test while 52 RT-PCR negative patients (28.1%) were declared positive based on their CT scan. 95 patients (51.3%) were tested negative and had normal chest 3D reconstruction.

57% of Covid-19 patients had a mild form, 15.5% had a medium form, 16.3% had a severe form, and 10% were critical (Table 1).

After the raw data computerized processing, 3D reconstructions of suspected COVID-19 patients' chests were done using several CT images. This made the 3D volume resolution better and the identification of the inflamed tissues in the patients’ lungs easier. To evaluate the efficiency of 3D reconstruction, five patients are presented in this paper. The first and second cases are covid-19 negative patients (Patient A1 and Patient A2), the third and fourth cases are PCR positive patients (Patient B1 and Patient B2), and the fifth case is a PCR false negative patient (Patient C).

### 3.1 Case (A): 3D reconstruction of a negative RT-PCR patients with normal CT images

#### 3.1.1 Patient A1

A 30-year-old man, health worker within the COVID-19 Isolation Department, showed up complaining of shortness of breath, headaches, fatigue and tachycardia for 8 days. The patient had no significant history.

Physical examination did not reveal any abnormalities, and rectal temperature was 37.1° Celsius.

Standard laboratory tests were all within normal limits, C-reactive protein level (0.5 mg/L; normal level, < 5 mg/L) and Ferritin level (246.4 ng/ml; normal level 23–300 ng/ml), D-dimer at 195 ng/ml (normal level 0–500 ng/ml) and no lymphopenia.

**Figure 2:**
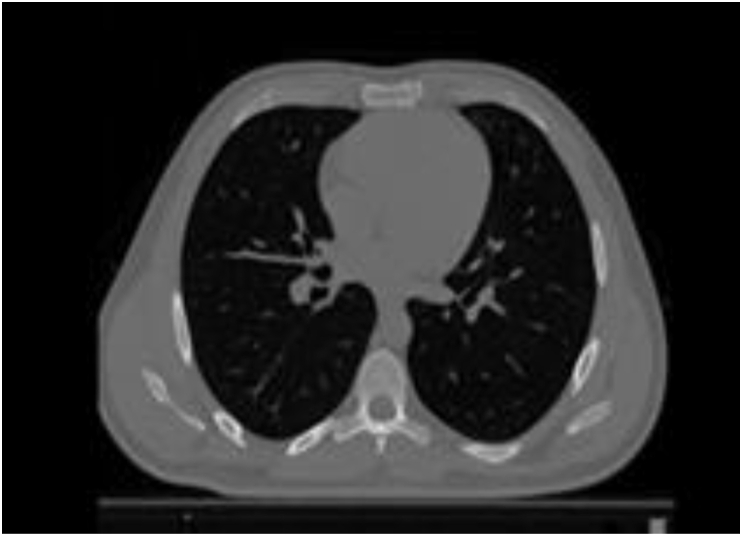
Chest CT slice of patient (A1) with no abnormalities.

The patient underwent unenhanced chest CT (Figure 2) for a suspicion of COVID-19 infection.

**Figure 3:**
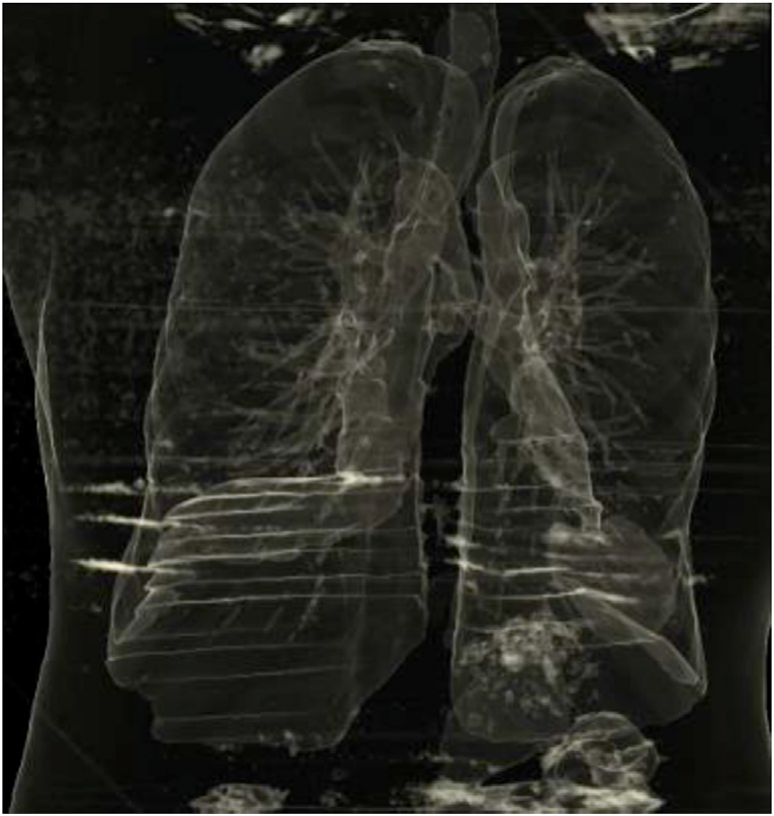
Image of the 3D reconstruction (A1): no abnormalities suggesting lung injuries related to pneumonia.

3D reconstruction showed no particular abnormalities (Figure 3).

#### 3.1.2 Patient A2

A 37-year-old woman, showed up with mild dyspnea, dry cough, headaches, and diarrhea. She was treated for iron deficiency anemia.

Physical examination revealed wheezing, and rectal temperature was 38.1° Celsius.

Standard laboratory tests suggested an infectious syndrome, C-reactive protein level (22 mg/L; normal level, < 5 mg/L) and Ferritin level (187.6 ng/ml; normal level 23–300 ng/ml), D-dimer at 31 ng/ml (normal level 0–500 ng/ml) and white blood count (12,245 per microliter, normal level (4,500–11,000/microliter).

**Figure 4:**
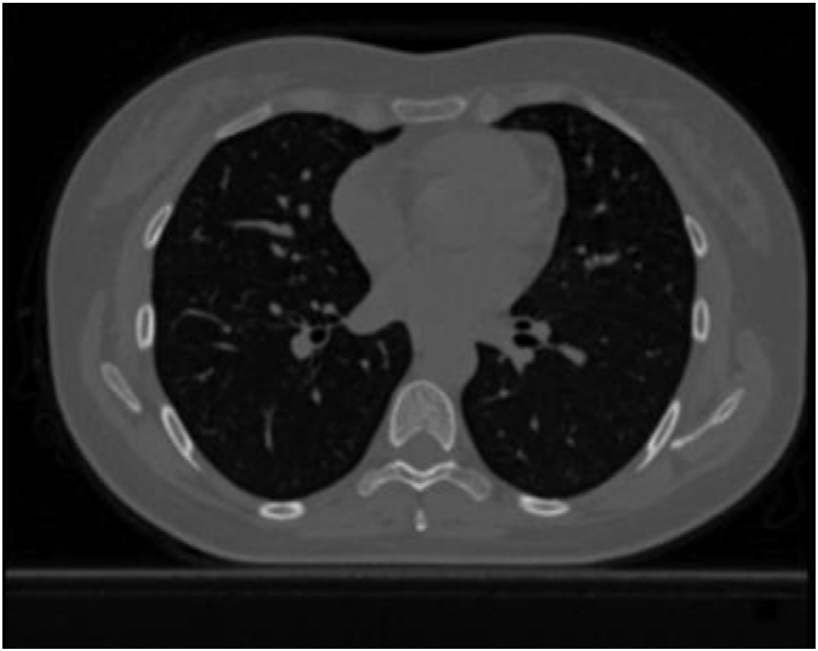
Chest CT slice of patient (A2) with no abnormalities.

The patient underwent unenhanced chest CT (Figure 4) for a suspicion of COVID-19 infection.

**Figure 5:**
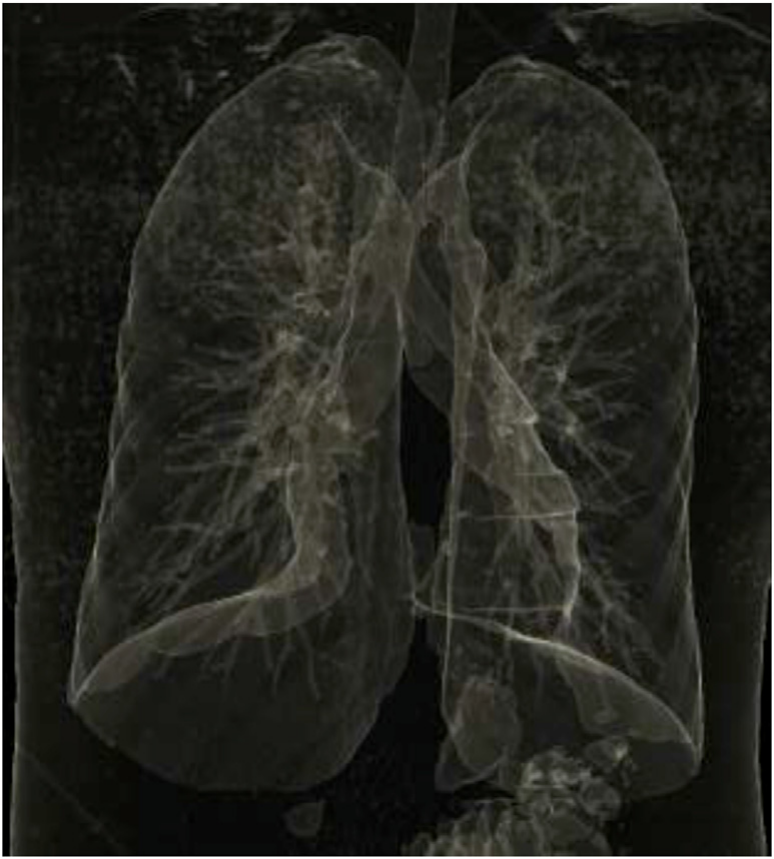
Image of the 3D reconstruction (A2): no abnormalities suggesting lung injuries related to pneumonia.

3D reconstruction showed no particular abnormalities (Figure 5).

### 3.2 Case (B): 3D reconstruction of a positive RT-PCR patient with abnormal CT images

#### 3.2.1 Patient B1

A 63-year-old women, under hypertension treatment, who had contact with a covid-19 positive patient, was admitted for suspected covid-19viral pneumonia. The symptoms started 8 days prior to admission, and consisted mainly of dry cough, sore throat, and a moderate dyspnea. Physical examination revealed a forehead temperature at 38.9 °C, dysphonia and a significant cough.

**Figure 6:**
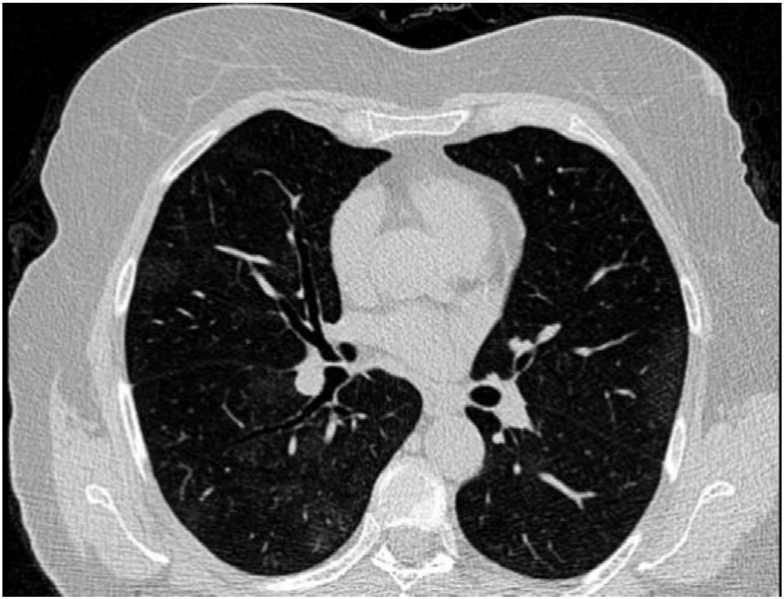
Chest CT slice of PCR positive patient (B1) with multilobar images of ground glass opacity (GGO) mainly located in the inferior lobes.

Routine laboratory values were mostly within limits with the exception of ferritinemia at 900.95 ng/ml, D-dimer test at 2556 ng/ml. Nasopharyngeal swab was positive for COVID-19 through real-time polymerase chain reaction (RT-PCR) confirming the diagnosis of COVID-19 pneumonia. A chest CT scan (Figure 6) was performed to look for abnormalities or complications.

**Figure 7:**
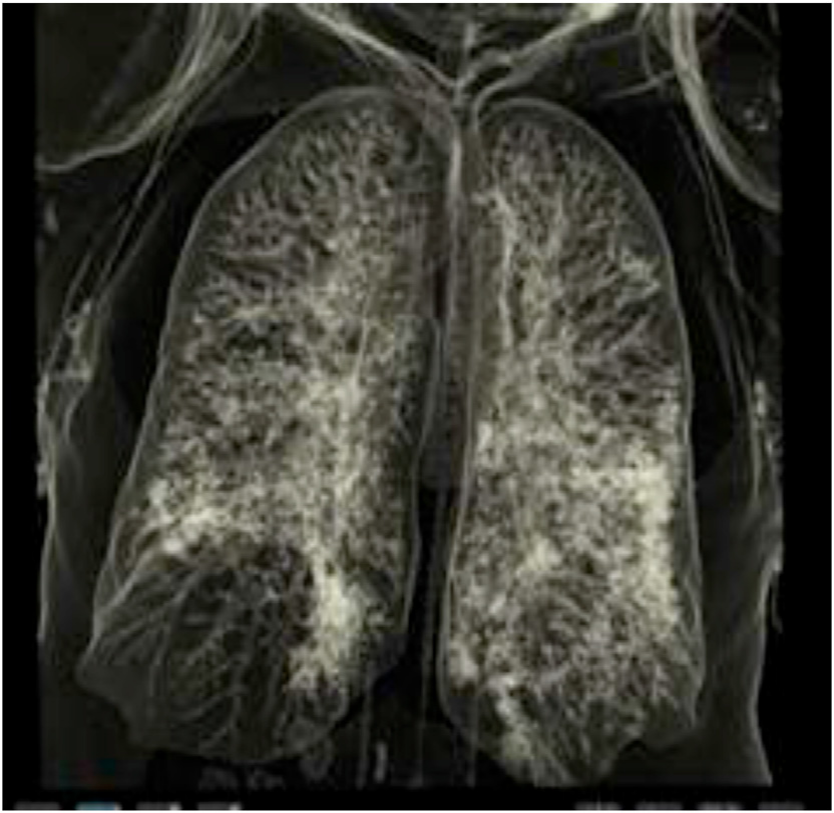
Images of the 3D reconstruction of PCR positive patient (B1) lungs showing enlarge injury of both lungs.

3D reconstruction (Figure 7) showed multilobar images of ground glass opacity (GGO) mainly located in the inferior lobes, but which did not spare the subpleural regions. Subpleural bands condensations were observed mainly in the lingula lobe. The patient was classified CORADS6 (according to CO-RADS radiology classification).

#### 3.2.2 Patient B2

A 69-year-old women, with underlying diabetes type 2 condition, and whose sister was tested positive, was admitted for suspected covid-19viral pneumonia. The symptoms started 2 days prior to admission, and consisted mainly of dyspnea, dizziness and fatigue. Physical examination revealed a rectal temperature at 38.4 °C and dry cough, in addition to respiratory struggle signs.

**Figure 8:**
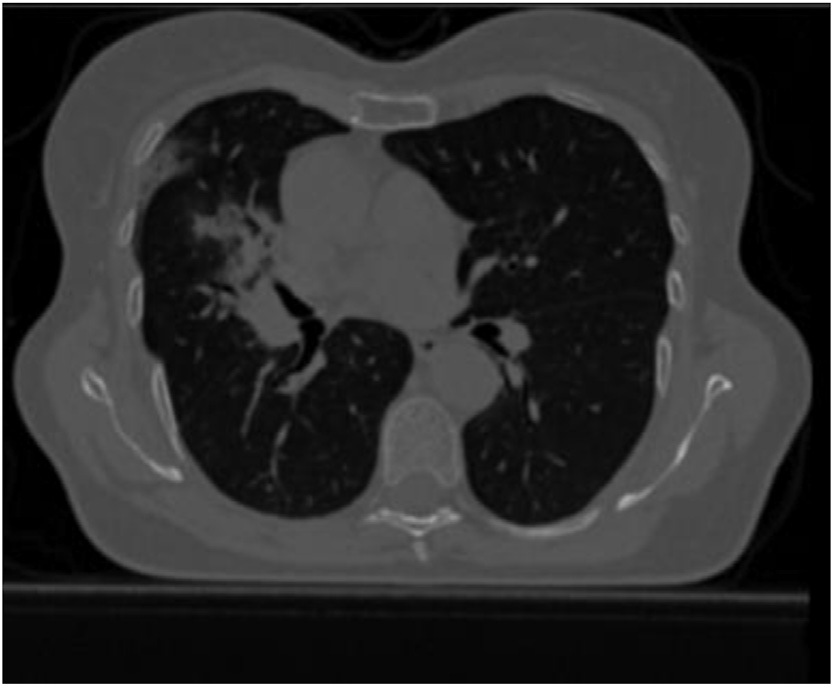
Chest CT slice of PCR positive patient (B2) showing GGO in right upper lobe and consolidation opacity in the right middle lobes.

Laboratory values were unusual: ferritinemia at 1405.95 ng/ml, D-dimer test at 2875 ng/ml. Nasopharyngeal swab was positive for COVID-19 through real-time polymerase chain reaction (RT-PCR) confirming the diagnosis of COVID-19 pneumonia. A chest CT scan (Figure 8) upon admission was performed to look for abnormalities or complications, and showed a GGO on the right upper posterior lobe and consolidation images on the right middle lobes.

**Figure 9:**
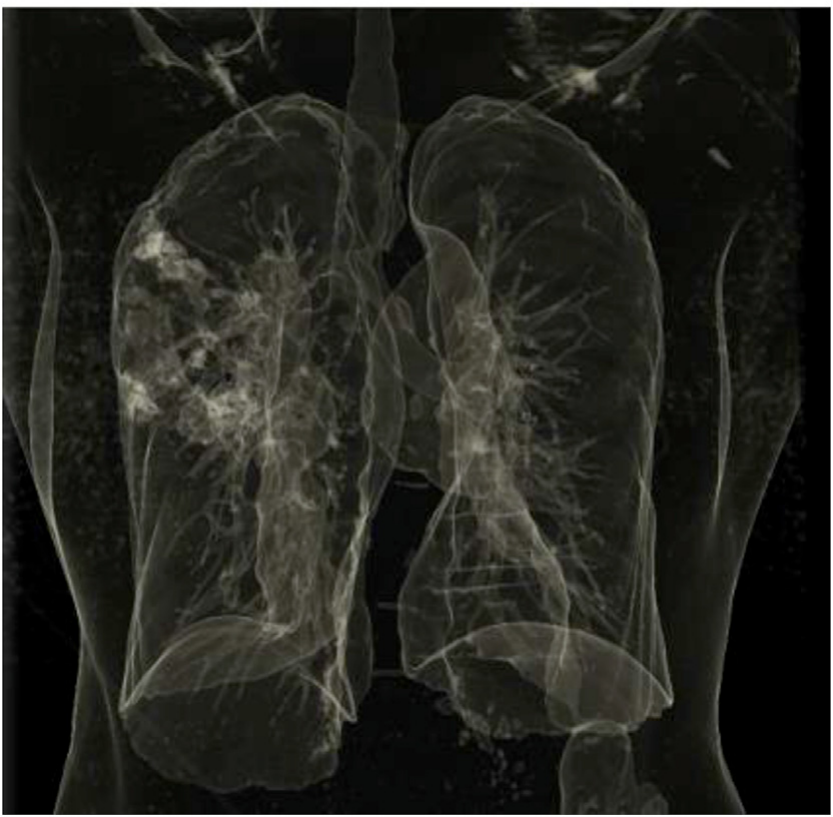
Image of the 3D reconstruction of PCR positive patient (B2) lungs showing limited injury of right lung and increased density in left middle lobes.

3D reconstruction (Figure 9) showed a limited damage in the right middle and upper posterior lobes, with a suspicious increased density in the left middle lobes. The patient was classified CORADS 4 (according to CO-RADS radiology classification) then CORADS 6 after PCR results.

### 3.3 Patient (C): 3D reconstruction of a negative RT-PCR patient with abnormal CT images

**Figure 10:**
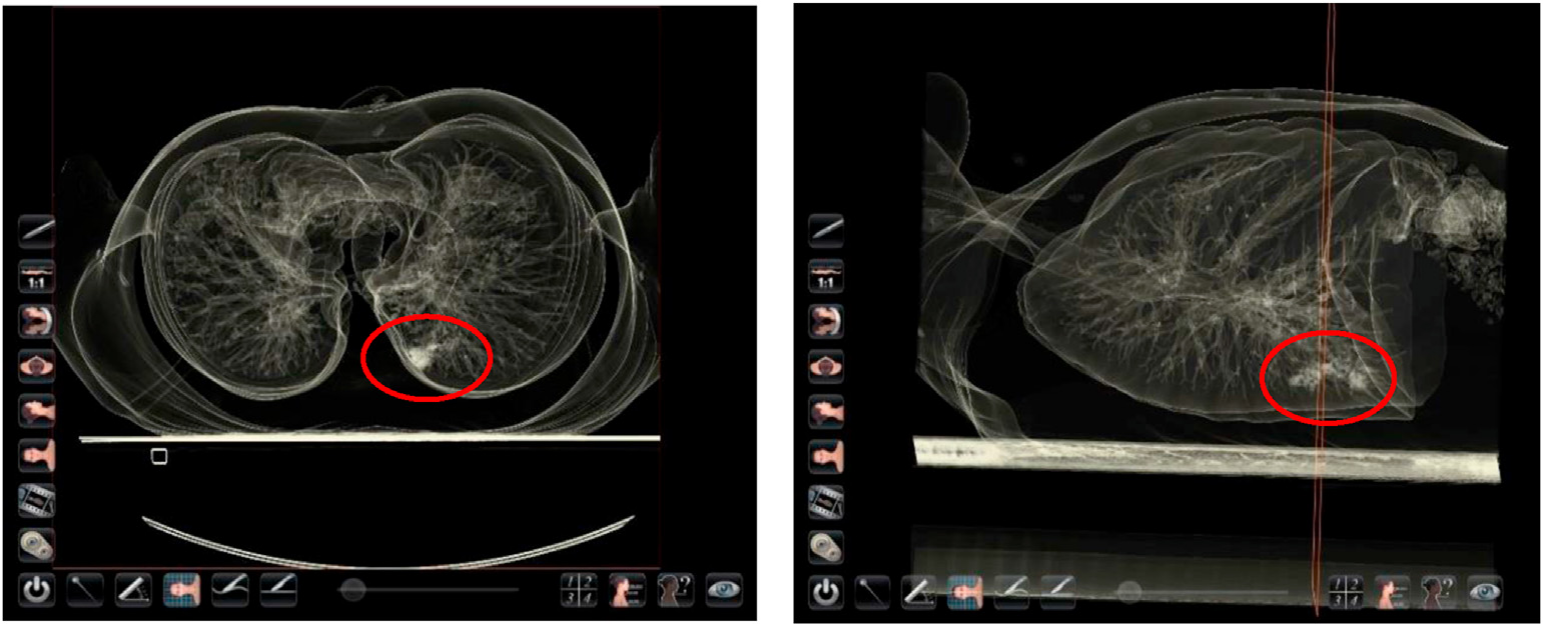
Images of the 3D reconstruction of PCR false negative patient (C), showed peripheral, bilateral, and basal predominant distributed areas of ground-glass opacities (outlined in red).

A 48-year-old man turned up complaining of fever. The detailed anamnesis analysis exhibited contact with a positive Covid-19 patient. Physical examination was normal, and forehead temperature was 38.4 °C. Routine laboratory values were within normal limits. 3D reconstruction (Figure 10) showed peripheral, bilateral, and basal predominant distributed areas of ground-glass opacities (outlined in red) with right lung predominance. This suggested diagnosis of novel coronavirus (COVID-19) pneumonia. RT-PCR testing on a nasopharyngeal swab came back negative 12 h after admission.

## 4 Discussion

### 4.1 Background of 3D reconstruction during the COVID-19 pandemic at cheikh zaid university hospital

The COVID-19 pandemic surprised Morocco by its rapid spread, like many other countries. Under these exceptional circumstances, and on a voluntary basis, healthcare teams were mobilized to deal with the large flow of patients. These teams did not necessarily have the required experience to assess the severity of the symptoms, nor to anticipate complications in the most vulnerable patients. There was an urgent need to identify rapid tools allowing a simple and effective understanding of the patient's clinical challenges. Especially with the difficulty to carry out a precise and complete clinical examination of a positive or highly suspect patient.

Cheikh Zaid Hospital being a university hospital, it resulted essential to involve volunteer medical students in establishing diagnosis and treatment, while ensuring their educational training with maximum safety and caution. The 3D reconstruction has proved its great interest in terms of diagnosis, prognosis and pedagogy.

### 4.2 3D reconstruction of COVID-19 CT scan

The reconstruction of the raw DICOM data has created a virtual volume that can be observed and studied. It was also possible to navigate in three dimensions or on a 2D plan in the reconstructed part. Generally, a low-quality reconstruction would be related to the processing applied to the raw data. It was not our case given the different algorithms implemented to improve our results. The use of a large number of thin slices made it possible to carry out calculations adapted to clinical and pedagogical purposes.

3D reconstruction of the lungs is more complex but also more promising in terms of raw data processing techniques. It allows us to obtain 3D unventilated lung volumes that are reliable and precise from a diagnostic point of view.

### 4.3 Correlation between 3D reconstruction and the clinical aspect

The 3D reconstruction on CT of the patients’ respiratory tract allowed a better apprehension and understanding of the symptomatology positive cases. Indeed, in the 05 cases explored in our article, the reconstruction helped anticipate the clinical evolution in a more or less precise way:

⁃ Patient (A1): negative PCR with normal CT images:

The clinical evolution matched the initial absence of abnormalities on the admission 3D reconstruction. Indeed, the patient's follow-up over a period of 14 days did not show any development of symptoms or deterioration of his general condition.

⁃ Patient (A2): negative PCR with normal CT images:

Patient was treated for gastroenteritis and the clinical evolution showed no signs of pneumonia. The dyspnea disappeared as soon as the patient received PCR results (which were negative) and was more related to anxiety.

⁃ Patient (B1): positive PCR withabnormal CT images:

The patient was classified as severe case based on his clinical condition and and the extensive distribution of condensations on the 3D reconstruction. He was admitted to the intensive care unit under oxygen therapy, and initiated of the therapeutic protocol 02 h after admission (awaiting PCR results). The symptomatology of the patient improved after 3 days of treatment, and disappeared on the 6th day, without the need for assisted ventilation.

⁃ Patient (B2): positive PCR withabnormal CT images:

The patient was classified as a mild COVID-19 case based on the clinical findings and the limited distribution and extension of the condensation on the 3D reconstruction. On the second day after admission, the patient presented a left lung murmur decrease, then went back to normal on the 7th day of treatment. The patient was declared cured on the 11th day of treatment.

⁃ Patient (C): negative PCR with CT abnormalities:

The patient was almost asymptomatic initially with normal lab test results. He was classified as a “moderate” case following 3D reconstruction. During clinical follow-up, on the 2nd day after admission, the patient experienced a moderate right basal chest pain, with a reduction of vesicular murmurs during routine auscultation and a decrease in the transmission of vibrations. A second nasopharyngeal sampling on the 9^th^ day of treatment finally came back positive. The immediate treatment following the results of the admission 3D reconstruction made it possible to keep the symptomatology to a moderate degree. The symptoms disappeared on the 4th day of therapeutic protocol.

We decided to make a 3D reconstruction on CT images taken after 10 days of treatment and before discharging the patient. The results made it easier for healthcare teams to observe the decrease of the condensations’ volume, as this decrease was only visible for experimented radiologists.

**Figure 11:**
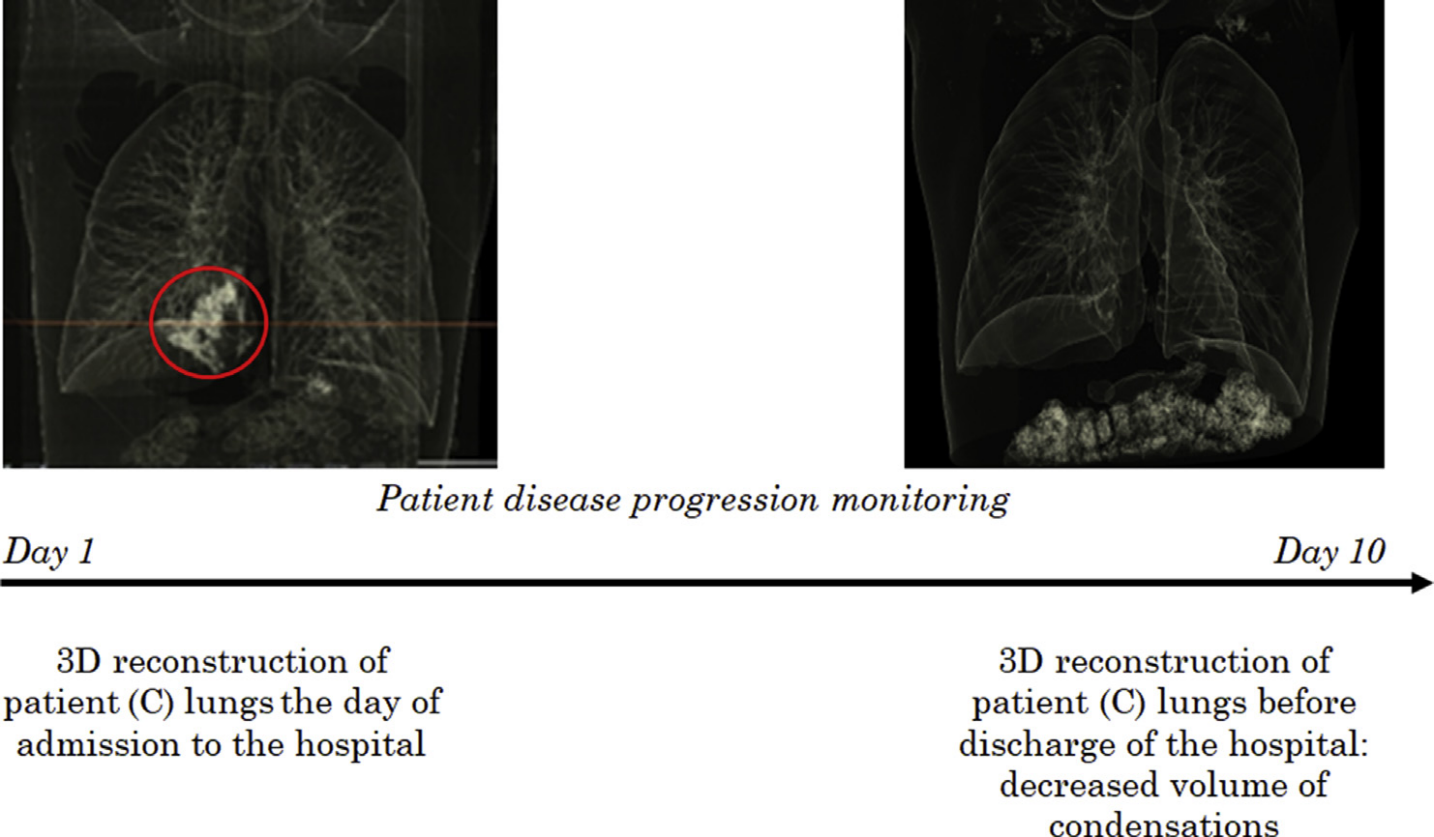
Evolution of Covid-19 disease from the day of patient's admission to the day of discharge.

The 3D reconstruction on chest CT images taken on the patient discharge day showed a regression in the volume of the condensations (Figure 11).

In the cases (A) and (B), the 3D reconstruction participated in establishing a clinical prognosis and speeding up the therapeutic decision. It also made it easier for our team to understand the severity of the viral pneumonia and its evolution. The use of 3D reconstruction on a wider series of patients (with statistical comparison of three-dimensional reconstruction results and clinical prognoses between subjects with the same CORADS grade) is necessary for a more objective demonstration of the correlation links.

## 5 Conclusion

Our study is innovative and relevant to help general medical staff and clinicians. In particular to improve the management of COVID-19 suspected patients. The results we present suggest that the integration of 3D CT reconstruction into routine work of clinicians should improve the speed and reliability of diagnosis. Consequently, it would increase the cure rate as well as reduce mortality and complications related to COVID-19. The limit of our method is that the 3D reconstruction is carried out using a closed-source software with limited documentation. The use of this software on several occasions has required to master its functionalities. However, due to the shortage of specialists involved in COVID-19, the general practitioners and the nurses were able to use the 3D reconstruction to assess the severity of the lung damage.

## Declarations

### Author contribution statement

Mouad Hasni & Zineb Farahat: Conceived and designed the experiments; Performed the experiments; Wrote the paper.

Azar Abdeljelil: Conceived and designed the experiments; Performed the experiments.

Kamal Marzouki: Analyzed and interpreted the data; Wrote the paper.

Mohamed Aoudad & Zakaria Tlemsani: Analyzed and interpreted the data.

Kawtar Megdiche: Conceived and designed the experiments; Contributed reagents, materials, analysis tools or data.

Nabil Ngote: Conceived and designed the experiments; Wrote the paper.

### Funding statement

This research did not receive any specific grant from funding agencies in the public, commercial, or not-for-profit sectors.

### Competing interest statement

The authors declare no conflict of interest.

### Additional information

No additional information is available for this paper.
